# The Effect of Surfactant-Modified Montmorillonite on the Cross-Linking Efficiency of Polysiloxanes

**DOI:** 10.3390/ma14102623

**Published:** 2021-05-17

**Authors:** Monika Wójcik-Bania, Jakub Matusik

**Affiliations:** Faculty of Geology, Geophysics and Environmental Protection, AGH University of Science and Technology, al. Mickiewicza 30, 30-059 Krakow, Poland; jmatusik@agh.edu.pl

**Keywords:** organo-smectite, montmorillonite, polymer clay nanocomposites, polysiloxane, hydrosilylation, quaternary ammonium salt

## Abstract

Polymer–clay mineral composites are an important class of materials with various applications in the industry. Despite interesting properties of polysiloxanes, such matrices were rarely used in combination with clay minerals. Thus, for the first time, a systematic study was designed to investigate the cross-linking efficiency of polysiloxane networks in the presence of 2 wt % of organo-montmorillonite. Montmorillonite (Mt) was intercalated with six quaternary ammonium salts of the cation structure [(CH_3_)_2_R’NR]^+^, where R = C_12_, C_14_, C_16_, and R’ = methyl or benzyl substituent. The intercalation efficiency was examined by X-ray diffraction, CHN elemental analysis, and Fourier transform infrared (FTIR) spectroscopy. Textural studies have shown that the application of freezing in liquid nitrogen and freeze-drying after the intercalation increases the specific surface area and the total pore volume of organo-Mt. The polymer matrix was a poly(methylhydrosiloxane) cross-linked with two linear vinylsiloxanes of different siloxane chain lengths between end functional groups. X-ray diffraction and transmission electron microscopy studies have shown that the increase in d-spacing of organo-Mt and the benzyl substituent influence the degree of nanofillers’ exfoliation in the nanocomposites. The increase in the degree of organo-Mt exfoliation reduces the efficiency of hydrosilylation reaction monitored by FTIR. This was due to physical hindrance induced by exfoliated Mt particles.

## 1. Introduction

Montmorillonite (Mt) is the best-known member of a smectite group of clay minerals and the most commonly used layered aluminosilicate for the preparation of clay–polymer nanocomposites. This is due to its high availability, low cost, capacity for swelling, and ion exchange properties. Moreover, Mt has a large surface area and high average aspect ratio (length-to-diameter ratio), good mechanical strength, and can undergo delamination [1,2,3]. Pristine montmorillonite is hydrophilic in nature due to the presence of hydrated inorganic cations e.g., Na^+^, Ca^2+^, or Mg^2+^. Thus, it is miscible only with hydrophilic polymers e.g., poly(ethylene oxide) [4] or poly(vinyl alcohol) [5]. However, most of the polymers are hydrophobic. To increase the compatibility of the hydrophobic polymer matrix toward clay mineral nanofillers, a proper modification of the aluminosilicate surface through the use of organic surfactants is needed [2,6]. The intercalation increases the clay mineral’s interlayer space and leads to organophilization, which enables the penetration of the polymer chains into the interlayer space of Mt. Alkylammonium ions are most often used to modify the clay mineral surfaces, but other salts such as sulfonium and phosphonium can also be used [2,7,8,9]. The organic cations lower the surface energy of the clay mineral, improving the wetting with the polymer matrix. Covalent organic functionalization of layered surface called silylation or silanization is also used to improve clay mineral compatibility with polymers [10].

Polysiloxanes, which belong to the group of inorganic polymers, are also used to prepare polymer–clay nanocomposites [2]. Polysiloxanes are attractive matrices for layered silicates due to their unique combination of properties [11]. The chain of polysiloxanes is composed of -Si-O- bridges, and the presence of substituents only at the silicon atom, i.e., at the second atom in the chain, affects the flexibility of the polymers’ chain. Moreover, these polymers exhibit a very low glass transition temperature (Tg), high chemical and thermal stability, low surface energy, good optical clarity, and resistance to UV radiation.

Despite the unique properties of polysiloxanes, the amount of research regarding the preparation of polysiloxane–clay mineral nanocomposites is scarce and relatively small as compared to other polymers. The studies mainly concerned the preparation of these materials with the sol–gel method from polydimethylsiloxane [12,13,14,15,16,17,18] and thermal cross-linking of silicone elastomers containing vinyl groups [19]. Another method used to obtain polysiloxane–clay mineral nanocomposites involves cross-linking of poly(methylhydrosiloxane) with divinylbenzene using the hydrosilylation reaction [20]. Commercial organo-montmorillonites from the Cloisite^®^ group (Southern Clay Products) and unmodified clay minerals were mainly used as mineral nanofillers. Organo-Cloisites^®^ are montmorillonites intercalated with ternary or quaternary ammonium salts [21]. Methyl, benzyl, 2-hydroxyethyl, and/or hydrogenated tallow (65% C_18_, 30% C_16_, and 5% C_14_) substituents are attached to the nitrogen atom in various amounts, which allows obtaining organic derivatives of clay minerals with various d-spacings and different interlayer chemistry. However, to the best of our knowledge, there are no in-depth systematic studies in the literature concerning the impact of the chemical structure of surfactants used to modify montmorillonite on the preparation and properties of polysiloxane nanocomposites. Such research is essential, as shown in the study by Simons at al. [22], because it enables a better design of nanocomposite systems, in this case for free radical polymerized systems. The study examined the effect of the position of the ammonium group, the inclusion of a polymerizable group, and the length of the alkyl chain in the surfactant on the properties of polystyrene-montmorillonite nanocomposites.

The aforementioned hydrosilylation reaction, which consists of the catalytic addition of an Si-H bond to multiple bonds, is used to obtain three-dimensional polysiloxane networks [23]. The availability of various hydrogen- and vinylsiloxanes makes it possible to obtain materials with a controlled structure and differing in cross-linking level [24]. It is also known that the degree of cross-linking of the system can influence the thermal properties of polysiloxane networks [25]. Studies on clay–polymer nanocomposites have shown that the addition of a mineral nanofiller to the polymer matrix improves, among others, the thermal properties of the system [26].

So far, no studies have been conducted on the influence of introduced montmorillonite modified with various quaternary ammonium salts on the cross-linking efficiency of polysiloxanes. In our previous work, we showed that with the increase in the content of mineral nanofiller in the polysiloxane matrix, the rate and efficiency of the hydrosilylation reaction decreased [27]. It is important to investigate the effect of the type of organo-montmorillonite used on the efficiency of the hydrosilylation reaction. The conducted research will be helpful in designing and optimizing the synthesis of a new clay mineral–polysiloxane nanocomposite.

In the present work, montmorillonite intercalated with three quaternary ammonium salts of the cation structure [(CH_3_)_3_NR]^+^ where R = C_12_, C_14_, C_16_ was used. Additionally, three salts were used where one of the methyl substituents was replaced with a benzyl group, which should further enhance organo-Mt and polysiloxane matrix compatibility. The type of surfactants used was selected in such a way that there was a gradual increase in the interaction of the polymer matrix with the mineral nanoadditive. Polysiloxane networks were obtained from poly(methylhydrosiloxane) and linear vinylsiloxanes, which differed in the chain length between the end functional groups. The networks were obtained by means of a hydrosilylation reaction with an equal molar ratio of Si-H to Si-CH=CH_2_ groups. Organo-montmorillonite was introduced in the amount of 2 wt % in relation to the weight of the polysiloxane matrix. The efficiency of the hydrosilylation reaction was determined using Fourier transform infrared (FTIR) spectroscopy. The conducted research showed that the type of organo-montmorillonite used influences the degree of cross-linking of the studied systems.

## 2. Materials and Methods

### 2.1. Materials

The montmorillonite (Mt) from Wyoming deposit (USA) was provided by the Clay Minerals Society (structural formula (Ca_0.12_Na_0.32_K_0.05_)[Al_3.01_Fe(III)_0.41_Mn_0.01_Mg_0.54_ Ti_0.02_][Si_7.98_Al_0.02_]O_20_(OH)_4_). The elemental composition (wt %) of the Mt clay mineral determined by X-ray fluorescence is: SiO_2_ (62.5), Al_2_O_3_ (19.9), Fe_2_O_3_ (3.79), TiO_2_ (0.15), MgO (2.24), CaO (1.65), Na_2_O (1.35), K_2_O (0.67), others (0.66). The cation exchange capacity (CEC) of Mt determined by the hexamminecobalt (III) chloride adsorption method [28] is 75.35 ± 1.33 meq/100 g.

The alkyl ammonium surfactants for the modification were of analytical grade and purchased from Sigma-Aldrich (Saint Louis, MO, USA). Surfactants were used in the form of bromides: dodecyltrimethylammonium (C_12_), tetradecyltrimethylammonium (C_14_), hexadecyl-trimethylammonium (C_16_) and chlorides: benzyldimethyldodecylammonium (BC_12_), benzyldimethyltetradecylammonium (BC_14_), benzyldimethylhexadecylammonium (BC_16_).

Poly(methylhydrosiloxane) (P) (viscosity 35–45 cSt) with trimethylsiloxy groups at both ends of the polymer chain and cross-linking agents i.e., 1,3-divinyltetramethyldisiloxane (A) and poly(dimethylsiloxane) (B) terminated at both ends by vinyldimethylsiloxy groups (viscosity 4–8 cSt, molecular weight 770 g/mol) were provided by ABCR (Karlsruhe, Germany) and used in the experiments as received.

Platinum(0)-1,3-divinyl-1,1,3,3-tetramethyldisiloxane complex (Karstedt’s catalyst) solution in xylene (2 wt % Pt) was purchased from Sigma-Aldrich (Saint Louis, MO, USA) and applied as received.

### 2.2. Modification of the Montmorillonite

The montmorillonite was modified with alkyl ammonium surfactants following the stoichiometric proportion to its cation exchange capacity (1.0 CEC). The modification was carried out in accordance with the procedure described in detail in our earlier work [27]. The aqueous ammonium salt solution was added to the aqueous dispersion of smectite (32 g/L). The resulting mixture was sonicated and then stirred for 24 h at 60 °C. After this time, the intercalated materials were washed with distilled water until the negative AgNO_3_ test proved the lack of Cl^−^ or Br^−^ ions in the solution. The last step was to disperse the organo-Mt in distilled water, sonicate, freeze in liquid nitrogen, and freeze-dry.

In the further part of the paper, for organo-montmorillonite, symbols will be used to denote the type of surfactant employed. For example, MtC_12_ denotes montmorillonite intercalated with dodecyltrimethylammonium bromide.

### 2.3. Preparation of Clay Mineral–Polysiloxane Nanocomposite

Two series of polysiloxane networks were prepared by hydrosililative cross-linking of poly(methylhydrosiloxane) with linear vinylsiloxanes i.e., cross-linker A and B, conducted at 60 °C in the presence of Karstedt’s catalysts. Equimolar amounts of reactive groups (Si–H and Si–CH=CH_2_) were applied. The amount of organo-montmorillonite introduced into the studied systems was set to 2 wt % with respect to the polysiloxane matrix. The initial reaction mixture with the addition of mineral nanofiller was subjected to sonication to facilitate the exfoliation and uniform distribution of the organo-Mt layers in the polymer matrix. The procedures and amounts of reagents described in the previous work [27] were used to obtain the studied materials.

Polysiloxane networks without the addition of nanofillers containing A or B cross-linker will be referred to as “PA” and “PB”, respectively. Clay mineral–polysiloxane nanocomposites will additionally contain the symbol of the organo-montmorillonite, for example PAMtC_12_.

### 2.4. Characterization Methods

Powder X-ray diffraction (XRD) patterns were recorded with a SmartLab 9.0 (Rigaku, Tokyo, Japan) diffractometer applying Ni-filtered Cu Kα (λ = 1.5406 Å) radiation, in the 2–75 range °2θ with constant step equal to 0.05°2θ. The diffractometer is equipped with a D/teX Ultra 250 silicon strip detector. The XRD patterns for raw and intercalated montmorillonite were recorded for powdered and not oriented samples. In the case of clay mineral–polymer composites, their dispersion in isopropyl alcohol was placed on a measuring plate (non-refection holder made of the single-crystal silicon), and the measurement was made after evaporation of the solvent.

The FTIR spectra were collected by Thermo Scientific Nicolet 6700 spectrometer (Thermo Fisher Scientific, Waltham, MA, USA) using the transmission mode in the 4000–400 cm^–1^ spectral range at 4 cm^–1^ resolution with 64 scans. A sample of the starting reaction mixture (i.e., polymer, cross-linker, and organo-montmorillonite after the sonication process and before adding the catalyst) was placed as a thin film on a KBr pellet. A standard pellet (1 wt % sample mixed with KBr) was prepared from the samples after 72 h of the reaction. Three analyses were performed for all the samples. Quantitative analysis of the recorded FTIR spectra was performed using the Omnic program. The integral intensity of the band due to Si-H at ≈2160 cm^−1^ and C-H bond from Si-CH_3_ groups at ≈1260 cm^−1^ were calculated. The efficiency of the hydrosilylation reaction was calculated as follows: (ratio of the integral intensity of the Si-H/Si-CH_3_ bands from the spectra of the samples after 72 h of the reaction)/(the ratio of the integral intensity of the Si-H/Si-CH_3_ bands from the spectra of the samples of the initial reaction mixture) × 100%.

The CHN elemental analysis was performed through the combustion of samples and the measurement of purified and separated gaseous products using the VarioEL III Elementar analyzer (Langenselbold, Germany). The surfactant content in organo-montmorillonite was calculated from nitrogen and carbon content, and the results were averaged. For the calculation based on the carbon content, the amount of carbon trace that was present in the raw montmorillonite sample was taken into account.

Textural parameters were derived from N_2_ adsorption/desorption measurements performed at −196 °C with the use of an ASAP 2020 (Micromeritics, Norcross, GA, USA) device. Prior to the measurement, each sample was vacuum-heated at 150 °C. Specific surface area (S_BET_) was calculated according to the Brunauer–Emmett–Teller (BET) method [29], in the relative pressure range 0.05–0.2. The total pore volume ($V_{tot}^{0.99})$ was determined from the amount of N_2_ adsorbed at p/p_0_ = 0.99. The volume of micropores ($V_{mic}^{DR}$) (d < 2 nm) was calculated, using the Dubinin–Radushkevich method according to the procedure described in the ISO 15901:3-2007 standard. The volume of mesopores ($V_{mes}^{BJH}$) (2 < d < 50 nm) was calculated, using the Barrett–Joyner–Halenda (BJH) equation [30]. The macropore volume ($V_{mac}$) was calculated using the following equation:

(1) $$V_{mac}=V_{tot}^{0.99}-\left(V_{mic}^{DR}+V_{mes}^{BJH}\right).$$

Transmission electron microscopy (TEM) investigations were performed on an FEI TECNAI TF 20 X-TWIN (FEG) transmission electron microscope (Hillsboro, OR, USA). Samples for TEM analyses were prepared by pouring a dispersion of the examined material in isopropyl alcohol onto a carbon film-coated copper grid, which was followed by evaporation of the solvent.

## 3. Results and Discussion

### 3.1. Characterization of the Raw and Intercalated Montmorillonite

**Figure 1:**
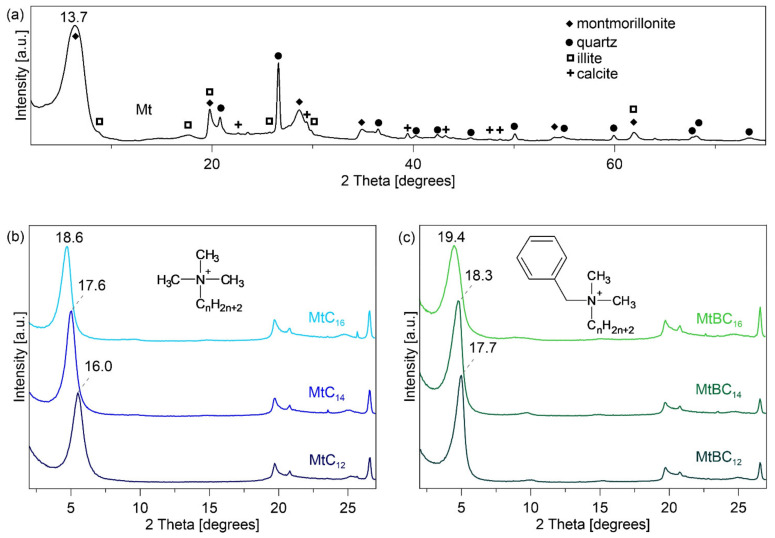
X-ray diffraction patterns of: (**a**) raw montmorillonite; (**b**) montmorillonite intercalated with alkyltrimethylammonium salts; (**c**) montmorillonite intercalated with benzyldimethyltammonium salts. Structure of alkylammonium ions (n—number of carbon atoms in alkyl chain, equal to 12, 14, and 16).

As mentioned above, montmorillonite was modified with quaternary ammonium salts with different alkyl chain lengths and with or without benzyl substituent group. The use of surfactants with such cationic structures was to allow the preparation of mineral materials with different interlayer distances and interlayer chemistry. It was assumed this will affect interactions between the organo-mineral nanoadditive and the polysiloxane matrix. X-ray diffraction was used to study the changes in the surface properties of a montmorillonite through analysis of the basal spacing of the raw and intercalated montmorillonite (Figure 1). The XRD pattern revealed the basal spacing value (d_001_) of the raw montmorillonite equal to 13.7 Å (Figure 1a). Additionally, the presence of an insignificant admixture of quartz, illite, and calcite in the sample was detected.

After the intercalation process with quaternary ammonium salts, an expansion of the montmorillonite layers was noticed (Figure 1b,c). Based on the basal spacing value (d_001_), the arrangement of alkylammonium ions in the interlayer space of smectites has been established [31]. The alkylammonium ions in MtC_12_, MtC_14_, and MtBC_12_ are arranged in monolayers (d_001_ ≈ 1.4 nm), while those in MtBC_14_, MtC_16_, and MtBC_16_ are arranged in bilayers (d_001_ ≈ 1.8 nm) with alkyl chain axes lying parallel to the silicate layers. In both the MtC_n_ and MtBC_n_ series (where n = 12, 14, 16), an increase in the d_001_ basal spacing is observed with increasing alkyl chain length. The obtained results are consistent with previous studies which showed a linear relationship between the increase of d-spacing and the mass ratio between introduced organic compounds and clay minerals [32]. It is also worth noting that the value of the basal spacing is influenced not only by the length of the alkyl chain but also by the presence of the benzyl substituent. The organo-montmorillonites containing benzyl groups have greater interlayer distances than their analogs without this substituent, e.g., MtBC_16_—19.4 Å and MtC_16_—18.6 Å. This is probably related to the arrangement of alkylammonium ions in the interlayer space of smectites. The ammonium groups are attached to the clay mineral surface, while the alkyl chains form a bilayer structure [31]. Replacing one methyl substituent at the quaternary nitrogen atom with a much larger benzyl substituent results in greater expansion of the adjacent clay mineral layers.

**Figure 2:**
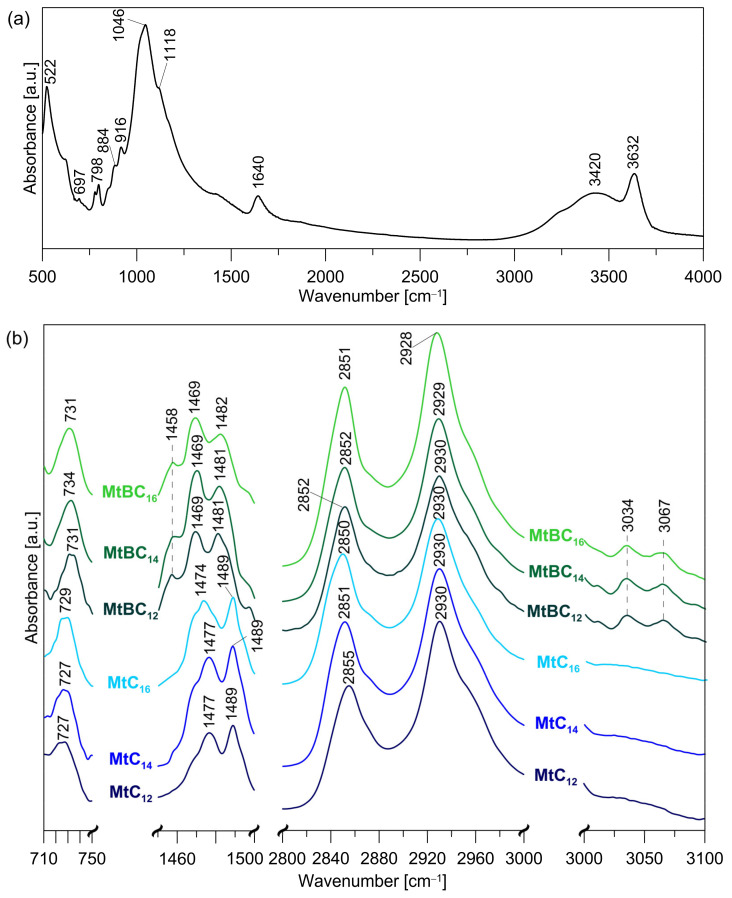
FTIR spectra: (**a**) raw montmorillonite; (**b**) montmorillonite intercalated with quaternary ammonium salts.

The sample of the raw montmorillonite and its modified organic derivatives were analyzed by infrared spectroscopy. In the FTIR spectrum of the raw montmorillonite (Figure 2a), the most intensive bands at 1046 and 522 cm^−1^ are attributed to Si-O in-plane stretching and Si-O bending vibrations, respectively [33,34]. Additionally, the shoulder at 1118 cm^−1^ originating from Si-O stretching out-of-plane can be observed. The broad bands centered at 3420 cm^−1^ and 1640 cm^−1^ are due to –OH stretching and bending vibrations of the interlayer water. Whereas the band at 3632 cm^−1^ is due to the –OH band stretch of structural Al-OH, the bands at 916 and 884 cm^−1^ are attributed to AlAlOH and AlFeOH bending vibrations, respectively. In the FTIR spectrum, the bands originating from the platy form of tridymite and quartz can also be observed at 798 and 697 cm^−1^, respectively [34].

The infrared spectra of montmorillonite samples modified with various ammonium salts reveal vibrational bands of organic modifiers. The intercalation did not cause any distortion of aluminosilicate structure. Selected ranges of FTIR spectra in which new bands are visible from the surfactant are shown in Figure 2b. The bands in the 2928–2930 and 2850–2855 cm^−1^ ranges are attributed to the CH_2_ asymmetric stretching mode (ν_as_ CH_2_) and the symmetric stretching mode (ν_s_ CH_2_), respectively [35,36,37,38]. The bands corresponding to the bending vibrations of C-H bonds in the methylene group occur in the ranges of 710–750 cm^−1^ (rocking mode ρ CH_2_) and 1450–1500 cm^−1^ (scissoring mode δ_s_ CH_2_). The spectra of materials modified with ammonium salts with a benzyl substituent (MtBC_n_ series) have additional bands indicating the presence of an aromatic ring. The band at 1458 cm^−1^ is due to skeletal vibrations involving C=C stretching within the ring [35,37]. Whereas, aromatic C-H stretching bands occur at the 3000–3100 cm^−1^ range. 

It is worth noting that the frequencies of the CH_2_ stretching absorption bands (ν_as_ CH_2_ and ν_s_ CH_2_) shifted toward higher frequencies as the length of the alkyl chains in the organo-Mt samples increased. The shifts can be attributed to the change of the alkyl chains conformation. The frequencies of the CH_2_ stretching bands of hydrocarbon chains are extremely sensitive to the conformational changes of the chains [39]. The narrow absorption bands appear at 2918 (ν_as_ CH_2_) and 2850 cm^−1^ (ν_s_ CH_2_) in the infrared spectra only when the chains are highly ordered (*trans* conformation) [40]. The frequencies of these bands shift upward as the average content of *gauche* conformers increases.

**Figure 3:**
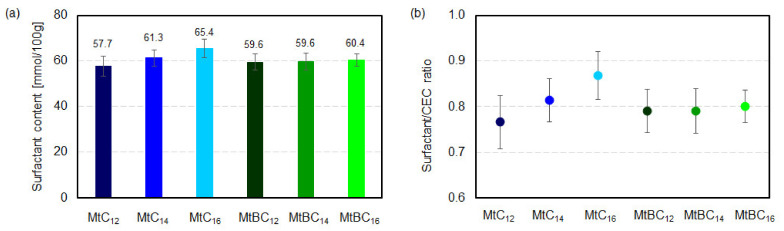
(**a**) Molar content of ammonium salts in organo-montmorillonite; (**b**) the ratio of surfactant in organo-montmorillonite to cation exchange capacity in raw montmorillonite.

The results of the elemental analysis were used to calculate the content of ammonium salts in the samples of modified montmorillonites (Figure 3a). The molar content of surfactants in the MtBC_n_ series is similar and amounts to approximately 60 mmol/100 g. While, in the MtC_n_ series, a slight increase in the molar content of ammonium salt is observed with the increase in the length of the carbon chain in organic molecues i.e., from 57.7 to 65.4 mmol/100 g. This is probably related to the decreasing water solubility of surfactants with increasing alkyl chain length, which makes it difficult to wash out completely from the system. Figure 3b shows the ratio of surfactant in organo-montmorillonite to cation exchange capacity of the raw montmorillonite. The obtained results indicate that the content of organic cations was lower than the CEC of raw montmorillonite and was at a similar level, i.e., in the range of 0.77–0.87.

**Figure 4:**
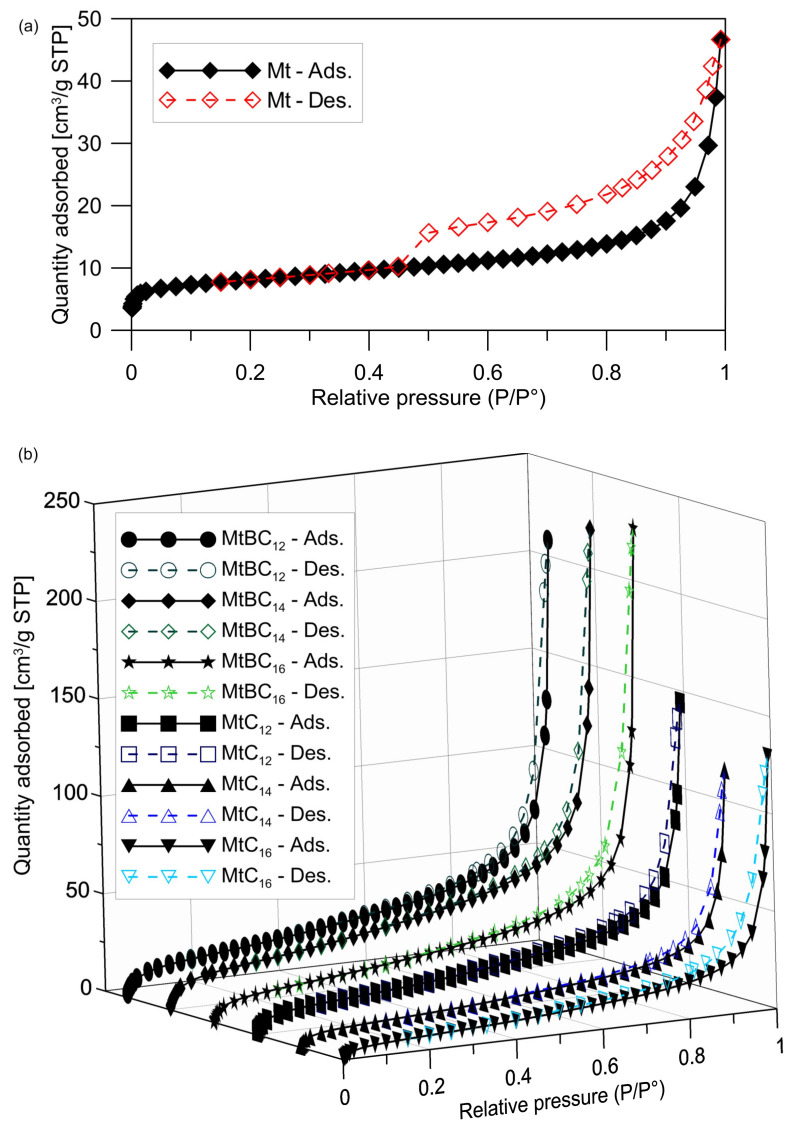
Comparison of N_2_ adsorption and desorption isotherms at −196 °C: (**a**) raw montmorillonite; (**b**) montmorillonite intercalated with quaternary ammonium salts.

Textural parameters also serve as supplementary data to verify the surface modification of montmorillonite by evaluating their specific surface area and pore volume changes after surfactant treatment. N_2_ adsorption/desorption isotherms obtained for the raw and organo-modified montmorillonite are presented in Figure 4. The shape of the isotherms corresponds to type IIB according to the IUPAC classification [41], refined by Rouquerol et al. [42], with H3 hysteresis loop characteristics for aggregates of plate-like particles giving rise to slit-shaped pores. The isotherm shape indicates that the studied materials contain three types of pores. The presence of micropores is indicated by increased nitrogen uptake at low P/P°. The occurrence of mesopores and macropores is confirmed by the presence of the hysteresis loop and nitrogen adsorption in the relative pressure range of 0.98–1.00, respectively. The course of the recorded isotherms is similar, however, for the organo-montmorillonite samples, a sharp increase in the amount of adsorbed gas was observed at pressures close to saturation (Figure 4b). This indicates an increase in the macropore volume in the samples.

The S_BET_ and V_tot_ of organo-montmorillonite samples increased compared to the raw montmorillonite (Table 1). The only inconsistency was observed for the MtC_16_ sample, which has a slightly lower S_BET_ compared to the starting clay mineral. The V_tot_ of the materials after intercalation with ammonium salts is greater by ≈2.5 to almost 5 times than in the raw clay mineral sample. The V_tot_ increase was mainly due to an increase in the volume of macro- and mesopores. The micropore volume in the MtC_n_ series samples is almost the same as in the raw montmorillonite and increases 2–2.5 times in the MtBC_n_ series. It is also worth noting that the percentage of micropore volume in organo-montmorillonite samples is much lower in relation to unmodified montmorillonite. The decrease in the percentage of mesopores does not exceed 9%, while a significant increase in the share of macropores in V_tot_ is observed. The ammonium benzyl modified montmorillonites have higher S_BET_ and V_tot_ than their MtC_n_ series counterparts. Additionally, for the MtBC_n_ series, an increase in V_tot_ is observed with increasing alkyl chain length.

Most studies have shown that the S_BET_ and V_tot_ of organo-montmorillonite decreased with an increase of the loaded surfactant amount [43,44]. Such S_BET_ reduction was also reported in study where ammonium salts were used for intercalation in amounts equal to 1.0 CEC [45]. This was explained by the fact that the large intercalated surfactants blocked the diffusion of N_2_. However, attention should be paid to the method of organo-montmorillonites’ drying after the modification process. In the works [43,44,45], the classic in-oven drying was used. While in the present work, the samples were dispersed in distilled water, frozen in liquid nitrogen, and lyophilized (Section 2.2). The use of such a drying procedure limited the aggregation of grains and the closing of meso- and macropores, because such phenomena may take place during classical drying. Organo-montmorillonite modified with nonionic octyl phenyl polyoxyethylene ether using a wet ball-milling method and then freeze-dried [46] also had a larger S_BET_ and V_tot_. The increase in these parameters was related to the pulverization of the massive particles during the wet ball-milling process. Another issue raised was the enlargement of the distance between stacked flakes of organo-montmorillonite, thus increasing the pore volume.

The nitrogen adsorption/desorption studies carried out in the present work have shown that the use of sonication and lyophilization in the preparation of organo-montmorillonites leads to the increase of the secondary (interparticle) porosity. The larger S_BET_ and V_tot_ of the organo-Mt should facilitate the interaction of the polymer matrix with the mineral nanofiller.

### 3.2. Monitoring of Cross-Linking Eficiency

**Figure 5:**
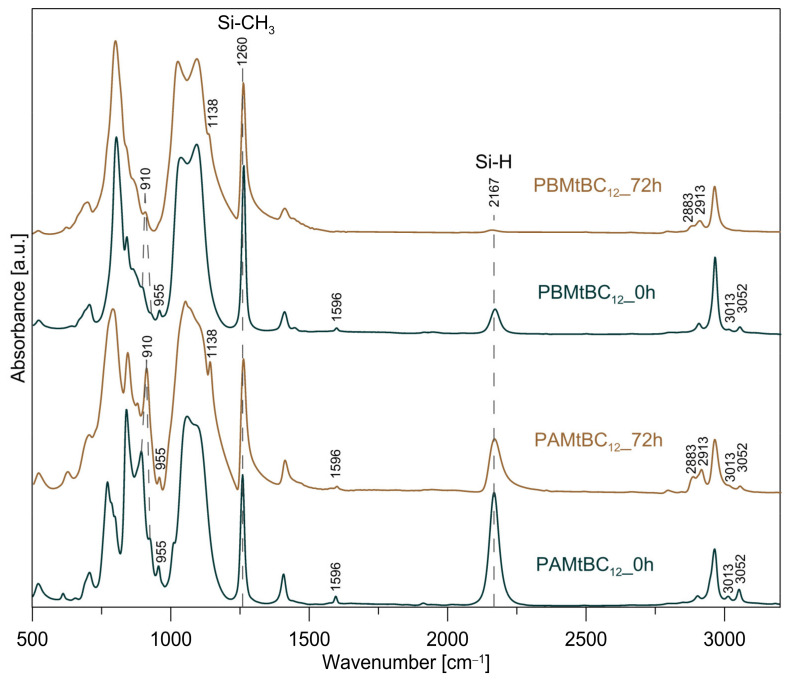
FTIR spectra of the starting and final reaction mixtures measured during cross-linking process.

FTIR spectroscopy is a convenient method used to determine the loss of Si-H groups from a system. It allows to monitor the course and efficiency of the hydrosilylation reaction [27,47]. It is also used to determine the consumption of Si-H groups during the reduction of transition metal ions [48,49]. Exemplary FTIR spectra of polysiloxane matrices containing 2 wt % of organo-mineral nanofiller are shown in Figure 5. These spectra were recorded for the initial reaction mixture and product formed after 72 h of reaction time. Positions and assignments of the main band in the spectra are described in detail in [27]. After the cross-linking process, a clear decrease in the intensity of the Si-H (2167, 892–924 cm^−1^) and Si–CH=CH_2_ (3052, 3013, 1596, and 955 cm^−1^) bands, which are involved in the hydrosilylation reaction, is observed. There are also new bands resulting from the formation of ‒CH_2_–CH_2_‒ linkages between Si atoms (2913, 2883, and 1138 cm^−1^).

**Figure 6:**
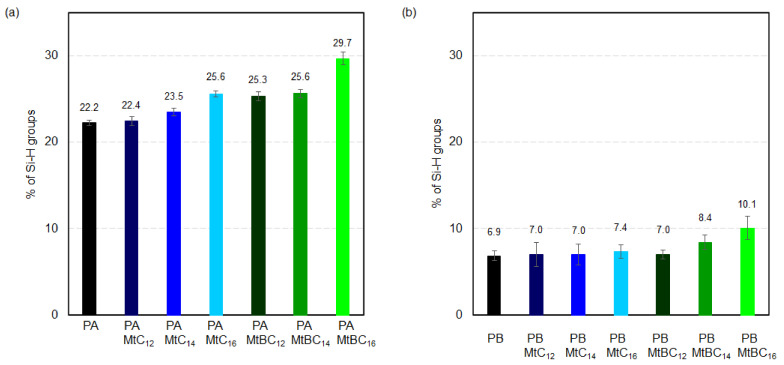
The efficiency of the hydrosilylation reaction is shown as the percentage of unreacted Si-H groups in the materials studied after 72 h of reaction: (**a**) PA series and (**b**) PB series.

As described in Section 2.4, the efficiency of the cross-linking reaction of the polysiloxane matrices in the presence of various organo-montmorillonites was determined from the ratio of the integral intensity of the Si-H to Si-CH_3_ bands in the starting reaction mixture and in the final product. The Si-CH_3_ bands do not participate in the hydrosilylation reaction, and their integral intensity does not change. For this reason, Si-CH_3_ bands can be used as an internal standard to which the changing integral intensity of the Si-H band relates. The results of quantitative analysis based on the recorded spectra are collected in Figure 6. The conducted studies have shown that the efficiency of the hydrosilylation reaction is influenced by the type of mineral nanofiller used. A greater effect is observed for the PA materials (Figure 6a), where a cross-linking agent with a shorter siloxane chain between the functional end groups was used. The presence in the polysiloxane matrix of the organo-montmorillonite MtC_12_, which has the lowest d_001_ basal spacing, i.e., 16.0 Å does not significantly affect the efficiency of the hydrosilylation reaction. On the other hand, an increasing d-spacing of the organo-Mt used leads to an increase in the amount of unreacted Si-H groups is observed. The obtained results suggest that an increase of the interlayer distance causes an increase in interactions between the organo-mineral nanoadditive and the polysiloxane matrix. Our earlier studies have shown that the lower efficiency of the hydrosilylation reaction of polysiloxane networks with introduced mineral nanofillers may be due to the presence of exfoliated clay mineral layers. This creates a physical obstacle and hinders the access of the cross-linking agent to the polymer chain [27].

The interaction between the organo-montmorillonite and the polysiloxane matrix is also influenced by the presence of a benzyl substituent in the surfactants used. It is worth paying attention to the hydrosilylation efficiency in the PAMtC_14_ and PAMtBC_12_ systems. The organo-montmorillonites present in these materials have almost the same d-spacing value, i.e., 17.6 Å and 17.7 Å for MtC_14_ and MtBC_12_ respectively, but the latter material has a benzyl substituent. The amount of unreacted Si-H groups in the PAMtBC_12_ system is about 2% higher than in the PAMtC_14_. This suggests that the presence of the benzyl substituent in the surfactant facilitates the interaction with the polysiloxane matrix. The largest amount of unreacted Si-H groups was observed for the PAMtBC_16_ sample, in which the mineral nanofiller has the largest basal spacing among the organo-Mts studied, and the surfactant used contained a benzyl substituent. The obtained results suggest that this system contains the most exfoliated clay layers, which hinder the further course of the hydrosilylation reaction, and hence, the reaction efficiency is the lowest.

In the PB series, only for the systems containing the following intercalates MtC_16_, MtBC_14_, and MtBC_16_, a visible increase in the amount of unreacted Si-H groups was observed (Figure 6b). The cross-linking efficiency of the PB matrix was much less affected by addition of organo-montmorillonites. This was probably due to the greater flexibility of the cross-linking agent B. The vinylsiloxane A is composed of two M′ units: (CH_3_)_2_(C_2_H_3_)SiO_0.5_, while in the B molecule between the M′ units, there is an additional siloxane chain consisting of eight D units: (CH_3_)_2_SiO. Larger distances between the end functional groups and subsequently less pronounced steric effects make them more accessible. As a result, they can react with more Si-H groups in the poly(methylhydrosiloxane) chain. This is confirmed by the results of the hydrosilylation reaction efficiency in systems without the mineral nanoadditives. The amount of unreacted Si-H groups in the PB system is about three times lower than in PA. If only partial organo-Mt exfoliation occurs in the polysiloxane matrix, longer and more flexible cross-linker molecules may bypass the individual clay mineral layers. Thus, their presence does not affect the cross-linking efficiency of the entire system. However, when the amount of exfoliated clay mineral layers in the polysiloxane matrix increases, their influence on the cross-linking process is already visible.

### 3.3. Characterization of Clay Mineral–Polysiloxane Nanocomposite—XRD and TEM Studies

**Figure 7:**
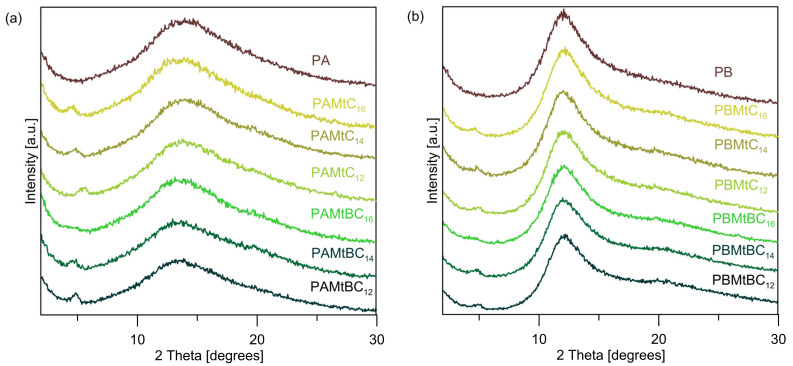
XRD patterns of the initial networks and clay mineral–polysiloxane nanocomposites: (**a**) PA series and (**b**) PB series.

XRD patterns of the initial polysiloxane networks without the addition of mineral nanofiller and clay mineral–polysiloxane nanocomposites are shown in Figure 7. The PA and PB matrices are amorphous materials. However, there is some ordering resulting from the arrangement of polymer chains and cross-linking molecules after the cross-linking process. In the PA diffraction pattern (Figure 7a), there is one broad reflection at 2θ ≈ 14°, while in the PB, two broad reflections are visible. The first one is more intense with a maximum at 2θ ≈ 12° and the other is a much less intense one centered at 2θ ≈ 20° (Figure 7b). It should be emphasized that the diffractograms of uncross-linked poly(dimethylsiloxane) and poly(methylhydrosiloxane) also contain two broad reflections [50,51]. One broad reflection in the PA networks is probably due to an overlap of the two mentioned XRD peaks.

The XRD patterns of the studied clay mineral–polysiloxane nanocomposites revealed the presence of the characteristic peak of the organo-montmorillonite corresponding to the (0 0 1) planes at 2θ ≈ 5° (Figure 7). Only in the matrices with MtBC_16_ as a mineral nanoadditive was this reflection not found. The obtained results indicate that only in the PAMtBC_16_ and PBMtBC_16_ systems, the organo-montmorillonite was completely exfoliated. In the rest of the materials, the stacking of 2:1 aluminosilicate layers along the c axis was preserved, which gives clear reflections on the recorded diffraction patterns. It is also worth noting that the lower intensity of organo-Mt XRD peaks in the PB series compared to PA is probably related to the greater intensity of the broad reflection at 2θ ≈ 12° coming from the PB matrix itself.

**Figure 8:**
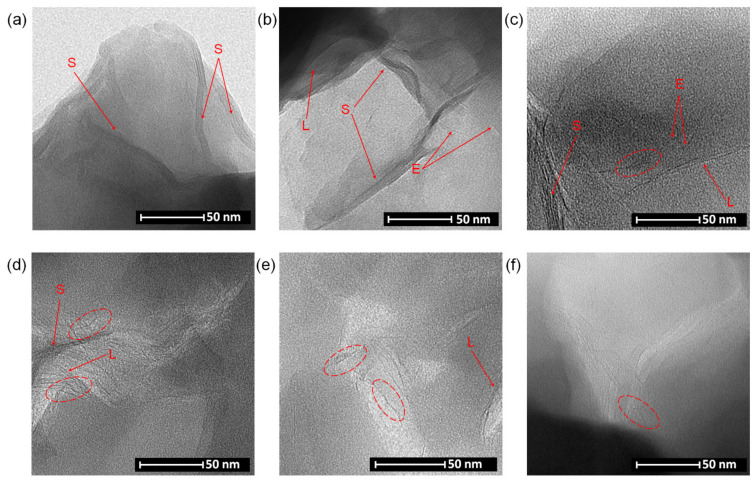
TEM images of organo-montmorillonite dispersed within a polysiloxane matrix: (**a**) PAMtC_12_, (**b**) PBMtC_16_, (**c**) PAMtBC_14_, (**d**) PBMtBC_14_, (**e**) PAMtBC_16_, and (**f**) PBMtBC_16_. (The *house of cards* structures and clay mineral layers (S-stacks of a few clay mineral layers, E-single exfoliated layer, L-stacks of two to three clay mineral layers) are marked with red ovals and arrows, respectively.).

XRD is a very useful method for characterizing the dispersion state of the organo-Mt in the polymer matrix and its extent; however, it does not give a complete picture of the degree of exfoliation of organo-Mts. TEM is a complementary method to XRD analysis, which provides additional information on the dispersion state of the clay mineral particles in the polymeric matrix. Selected TEM micrographs of the obtained nanocomposites are presented in Figure 8. In polysiloxane matrices, containing the organo-Mt with the smallest basal spacing, i.e., MtC_12_, no exfoliation occurred, and only stacks of a few clay mineral layers are present (Figure 8a). As the d-spacing of the organo-Mt increases, stacks of two to three clay layers and single exfoliated layers are also visible next to the stacked aluminosilicate layers (PBMtC_16_, Figure 8b). In the PAMtBC_14_ and PBMtBC_14_ systems (Figure 8c,d), a much larger number of individual clay mineral layers and associated structures resembling *house of cards*, i.e., rebonded exfoliated single layers, were observed [52]. It is worth noting that these systems still show non-exfoliated stacked 2:1 layers that were registered in the XRD diffractograms. It is also worth comparing the TEM images of the PBMtC_16_ and PBMtBC_14_ systems (Figure 8b,d). The MtC_16_ has a greater d-spacing than the MtBC_14_; however, in a system where montmorillonite intercalated with a benzyl surfactant was used, organo-Mt exfoliation occurred to a greater extent. According to the XRD results, the exfoliation process was most effective in the systems containing MtBC_16_. The TEM images (Figure 8e,f) show well-dispersed and disoriented single clay mineral layers. These matrices also contain a *house of cards* structures and a few stacks of two to three clay mineral layers.

As described in Section 3.2, the efficiency of the cross-linking reaction of polysiloxane matrices depends on the type of the mineral nanofiller. The XRD and TEM studies confirmed that the obtained clay mineral–polysiloxane nanocomposites differ in the degree of organo-montmorillonite exfoliation in the polymer matrix. The increase in the interlayer distance in the organo-Mt and the presence of a benzyl substituent in the surfactant used for intercalation results in greater interaction with the polymer matrix. Thus, the amount of partially exfoliated stacked silicate layers and individual clay mineral layers increased. The increase in the degree of exfoliation of the studied organo-Mt in the polysiloxane matrix causes the appearance of physical obstacles that limit the availability of functional groups involved in the hydrosilylation reaction, thus reducing the efficiency of this reaction.

As mentioned in the Introduction, organo-Mt has been introduced into the polymer matrix, for the purpose of, among others, improving its thermal properties. However, it should be noted that the thermal properties of polysiloxane networks were influenced by the degree of their cross-linking. Our research has shown that the presence of a mineral nanofiller reduces the efficiency of the hydrosilylation reaction and thus the degree of cross-linking of the entire polysiloxane matrix. Therefore, the addition of organo-Mt may deteriorate and not improve the thermal properties of clay mineral–polysiloxane nanocomposites. However, further studies are needed to verify this.

When designing new clay mineral–polysiloxane nanocomposites, obtained using the hydrosilylation reaction, the flexibility of hydrogen- and vinylsiloxanes molecules should be taken into account. The distances between the functional groups in these compounds should be large enough so that the presence of individual clay mineral layers does not hinder the cross-linking reaction taking place with the participation of these groups.

## 4. Conclusions

The study investigated the effect of montmorillonite modified with various quaternary ammonium salts on the cross-linking efficiency of polysiloxanes. The obtained organo-Mts had different basal spacings. The value of d-spacing was influenced by the different lengths of the alkyl substituents (C_12_, C_14_, and C_16_) as well as the presence or absence of a benzyl substituent in the surfactants used. The materials’ treatment procedure used after intercalation, i.e., sonication, freezing in liquid nitrogen, and lyophilization, caused an increase of the secondary (interparticle) porosity. Textural studies showed an increase in S_BET_ and V_tot_ of the obtained organo-Mts compared to pristine montmorillonite.

Polysiloxane matrices were obtained by cross-linking of poly(methylhydrosiloxane) with two linear vinylsiloxanes using the hydrosilylation reaction. The cross-linking agents used had different siloxane chain lengths between the functional end groups, which resulted in a different degree of cross-linking of the polymer matrices. The increase in the degree of organo-Mt exfoliation in the polysiloxane matrix decreased the efficiency of the hydrosilylation reaction. This was likely due to physical hindrances created by the exfoliated clay mineral layers limiting the access of the cross-linking agents to the Si-H groups in the polymer chains. Even a small degree of organo-Mt exfoliation increases the amount of unreacted Si-H groups in PA systems. In these systems, vinylsiloxane was used, with a shorter molecule and thus less mobile end functional groups. It should be emphasized that the presence of a benzyl substituent in organo-Mt facilitates the exfoliation process in the studied polysiloxane matrices.

It was demonstrated for the first time that an increase in the degree of organo-Mt exfoliation can affect the cross-linking efficiency of polysiloxane matrices depending on the cross-linking agents used. The obtained results will be helpful in optimizing the synthesis of new clay mineral–polysiloxane nanocomposites by selecting the appropriate components of the polymer matrix. In addition, systematic studies on the influence of the surfactant structure and the gradual increase in the compatibility of organo-Mt with the polysiloxane matrix will be useful in selecting the appropriate mineral nanofillers.

## Figures and Tables

**Table 1:** The textural parameters of raw and intercalated montmorillonites.

Sample	S_BET_ [m^2^/g]	$V_{tot}^{0.99}$ [cm^3^/g]	$V_{mic}^{DR}$ [cm^3^/g] (Share [%])	$V_{mes}^{BJH}$ [cm^3^/g] (Share [%])	$V_{mac}$ [cm^3^/g] (Share [%])
Mt	28.5	0.071	0.013 (18.3)	0.035 (49.3)	0.023 (32.4)
MtC_12_	47.7	0.221 (3.11) ^a^	0.015 (6.8)	0.112 (50.7)	0.094 (42.5)
MtC_14_	31.4	0.176 (2.48) ^a^	0.014 (8.0)	0.071 (40.3)	0.091 (51.7)
MtC_16_	26.9	0.196 (2.76) ^a^	0.012 (6.1)	0.091 (46.4)	0.093 (47.5)
MtBC_12_	63.0	0.314 (4.42) ^a^	0.023 (7.3)	0.141 (44.9)	0.150 (47.8)
MtBC_14_	71.6	0.334 (4.70) ^a^	0.034 (10.2)	0.157 (47.0)	0.143 (42.8)
MtBC_16_	53.6	0.346 (4.87) ^a^	0.027 (7.8)	0.139 (40.2)	0.180 (52.0)

^a^ ratio of $V_{tot}^{0.99}$ to $V_{tot}^{0.99}$ of raw montmorillonite.

## Data Availability

The data presented in this study are available on request from the corresponding author.

## References

[1] De Paiva L.B., Morales A.R., Valenzuela Díaz F.R. (2008). Organoclays: Properties, preparation and applications. Appl. Clay Sci..

[2] Alexandre M., Dubois P. (2000). Polymer-layered silicate nanocomposites: Preparation, properties and uses of a new class of materials. Mat. Sci. Eng. R Rep..

[3] Bhattacharya M. (2016). Polymer Nanocomposites-A Comparison between Carbon Nanotubes, Graphene, and Clay as Nanofillers. Materials.

[4] Aranda P., Ruiz-Hitzky E. (1992). Poly(ethylene oxide)-silicate intercalation materials. Chem. Mater..

[5] Yu Y.-H., Lin C.-Y., Yeh J.-M., Lin W.-H. (2003). Preparation and properties of poly(vinyl alcohol)–clay nanocomposite materials. Polymer.

[6] LeBaron P. (1999). Polymer-layered silicate nanocomposites: An overview. Appl. Clay Sci..

[7] Manias E., Touny A., Wu L., Strawhecker K., Lu B., Chung T.C. (2001). Polypropylene/Montmorillonite Nanocomposites. Review of the Synthetic Routes and Materials Properties. Chem. Mater..

[8] Zanetti M., Lomakin S., Camino G. (2000). Polymer layered silicate nanocomposites. Macromol. Mater. Eng..

[9] Madejová J., Barlog M., Jankovič Ľ., Slaný M., Pálková H. (2021). Comparative study of alkylammonium- and alkylphosphonium-based analogues of organo-montmorillonites. Appl. Clay Sci..

[10] Bee S.-L., Abdullah M.A.A., Bee S.-T., Sin L.T., Rahmat A.R. (2018). Polymer nanocomposites based on silylated-montmorillonite: A review. Prog. Polym. Sci..

[11] Mark J.E. (2004). Some interesting things about polysiloxanes. Acc. Chem. Res..

[12] Schmidt D.F., Clément F., Giannelis E.P. (2006). On The Origins of Silicate Dispersion in Polysiloxane/Layered-Silicate Nanocomposites. Adv. Funct. Mater..

[13] Lewicki J.P., Liggat J.J., Pethrick R.A., Patel M., Rhoney I. (2008). Investigating the ageing behavior of polysiloxane nanocomposites by degradative thermal analysis. Polym. Degrad. Stabil..

[14] Lewicki J.P., Liggat J.J., Patel M. (2009). The thermal degradation behaviour of polydimethylsiloxane/montmorillonite nanocomposites. Polym. Degrad. Stabil..

[15] Ma J., Xu J., Ren J.-H., Yu Z.-Z., Mai Y.-W. (2003). A new approach to polymer/montmorillonite nanocomposites. Polymer.

[16] Burnside S.D., Giannelis E.P. (2000). Nanostructure and properties of polysiloxane-layered silicate nanocomposites. J. Polym. Sci. B Polym. Phys..

[17] Burnside S.D., Giannelis E.P. (1995). Synthesis and properties of new poly(dimethylsiloxane) nanocomposites. Chem. Mater..

[18] Kirby R., Mosurkal R., Li L., Kumar J., Soares J.W. (2013). Polysiloxane-based Organoclay Nanocomposites as Flame Retardants. Polym. Plast. Technol..

[19] Anyszka R., Bieliński D.M., Pędzich Z., Szumera M. (2015). Influence of surface-modified montmorillonites on properties of silicone rubber-based ceramizable composites. J. Therm. Anal. Calorim..

[20] Segatelli M.G., Kaneko M.L.Q.A., Silva V.P., Yoshida I.V.P. (2014). Porous Ceramic Materials from Polysiloxane-Clay Composites. J. Brazil. Chem. Soc..

[21] Bumbudsanpharoke N., Ko S. (2019). Nanoclays in Food and Beverage Packaging. J. Nanomater..

[22] Simons R., Qiao G.G., Powell C.E., Bateman S.A. (2010). Effect of surfactant architecture on the properties of polystyrene-montmorillonite nanocomposites. Langmuir.

[23] Matisons J., Marciniec B. (2009). Hydrosilylation: A Comprehensive Review on Recent Advances.

[24] Nyczyk-Malinowska A., Wójcik-Bania M., Gumuła T., Hasik M., Cypryk M., Olejniczak Z. (2014). New precursors to SiCO ceramics derived from linear poly(vinylsiloxanes) of regular chain composition. J. Eur. Ceram. Soc..

[25] Colombo P., Mera G., Riedel R., Sorarù G.D. (2010). Polymer-Derived Ceramics: 40 Years of Research and Innovation in Advanced Ceramics. J. Am. Ceram. Soc..

[26] Alateyah A.I., Dhakal H.N., Zhang Z.Y. (2013). Processing, Properties, and Applications of Polymer Nanocomposites Based on Layer Silicates: A Review. Adv. Polym. Technol..

[27] Wójcik-Bania M. (2021). Influence of the addition of organo-montmorillonite nanofiller on cross-linking of polysiloxanes—FTIR studies. Spectrochim. Acta A.

[28] Ciesielski H., Sterckeman T., Santerne M., Willery J.P. (1997). Determination of cation exchange capacity and exchangeable cations in soils by means of cobalt hexamine trichloride. Effects of experimental conditions. Agronomie.

[29] Brunauer S., Emmett P.H., Teller E. (1938). Adsorption of Gases in Multimolecular Layers. J. Am. Chem. Soc..

[30] Barrett E.P., Joyner L.G., Halenda P.P. (1951). The Determination of Pore Volume and Area Distributions in Porous Substances. I. Computations from Nitrogen Isotherms. J. Am. Chem. Soc..

[31] Lagaly G., Ogawa M., Dékány I. (2013). Clay Mineral–Organic Interactions. Handbook of Clay Science.

[32] Paul D.R., Zeng Q.H., Yu A.B., Lu G.Q. (2005). The interlayer swelling and molecular packing in organoclays. J. Colloid Interf. Sci..

[33] Madejová J. (2003). FTIR techniques in clay mineral studies. Vib. Spectrosc..

[34] Tyagi B., Chudasama C.D., Jasra R.V. (2006). Determination of structural modification in acid activated montmorillonite clay by FT-IR spectroscopy. Spectrochim. Acta A.

[35] Silverstein R.M., Webster F.X., Kiemle D.J., Bryce D.L. (2014). Spectrometric Identification of Organic Compounds.

[36] Zhu J., He H., Zhu L., Wen X., Deng F. (2005). Characterization of organic phases in the interlayer of montmorillonite using FTIR and 13C NMR. J. Colloid Interf. Sci..

[37] Zope I.S., Dasari A., Yu Z.-Z. (2017). Influence of Polymer-Clay Interfacial Interactions on the Ignition Time of Polymer/Clay Nanocomposites. Materials.

[38] Slaný M., Jankovič Ľ., Madejová J. (2019). Structural characterization of organo-montmorillonites prepared from a series of primary alkylamines salts: Mid-IR and near-IR study. Appl. Clay Sci..

[39] Mendelsohn R., Brauner J.W., Gericke A. (1995). External infrared reflection absorption spectrometry of monolayer films at the air-water interface. Annu. Rev. Phys. Chem..

[40] Li Y., Ishida H. (2003). Concentration-Dependent Conformation of Alkyl Tail in the Nanoconfined Space: Hexadecylamine in the Silicate Galleries. Langmuir.

[41] Sing K.S.W. (1985). Reporting physisorption data for gas/solid systems with special reference to the determination of surface area and porosity (Recommendations 1984). Pure Appl. Chem..

[42] Rouquerol F., Rouquerol J., Sing K. (1999). Adsorption by Clays, Pillared Layer Structures and Zeolites. Adsorption by Powders and Porous Solids.

[43] He H., Zhou Q., Martens W.N., Kloprogge T.J., Yuan P., Xi Y., Zhu J., Frost R.L. (2006). Microstructure of HDTMA^+^-modified montmorillonite and its influence on sorption characteristics. Clay Clay Miner..

[44] Zhu J., Zhu L., Zhu R., Tian S., Li J. (2009). Surface microtopography of surfactant modified montmorillonite. Appl. Clay Sci..

[45] Shah K.J., Mishra M.K., Shukla A.D., Imae T., Shah D.O. (2013). Controlling wettability and hydrophobicity of organoclays modified with quaternary ammonium surfactants. J. Colloid Interf. Sci..

[46] Yan H., Zhang P., Chen X., Bao C., Zhao R., Hu J., Liu C., Lin Q. (2020). Preparation and characterization of octyl phenyl polyoxyethylene ether modified organo-montmorillonite for ibuprofen controlled release. Appl. Clay Sci..

[47] Hasik M., Wójcik-Bania M., Nyczyk A., Gumuła T. (2013). Polysiloxane–POSS systems as precursors to SiCO ceramics. React. Funct. Polym..

[48] Wójcik-Bania M., Stochmal E., Duraczyńska D. (2020). Silver nanoparticles deposited on polysiloxane networks as active catalysts in dye degradation. J. Appl. Polym. Sci..

[49] Wójcik-Bania M., Olejarka J., Gumuła T., Łącz A., Hasik M. (2014). Influence of metallic palladium on thermal properties of polysiloxane networks. Polym. Degrad. Stabil..

[50] Wójcik-Bania M., Łącz A., Nyczyk-Malinowska A., Hasik M. (2017). Poly(methylhydrosiloxane) networks of different structure and content of Si-H groups: Physicochemical properties and transformation into silicon oxycarbide ceramics. Polymer.

[51] Andrianov K.A., Slonimski G.L., Zhdanov A.A., Levin V.Y., Godovski Y.K., Moskalenko V.A. (1972). Some physical properties of polyorganosiloxanes. I. Linear polyorganosiloxanes. J. Polym. Sci. A 1.

[52] Okamoto M., Nam P.H., Maiti P., Kotaka T., Hasegawa N., Usuki A. (2001). A House of Cards Structure in Polypropylene/Clay Nanocomposites under Elongational Flow. Nano Lett..

